# The Ubiquitin Interacting Motif-Like Domain of Met4 Selectively Binds K48 Polyubiquitin Chains

**DOI:** 10.1016/j.mcpro.2021.100175

**Published:** 2021-11-09

**Authors:** Mark Villamil, Weidi Xiao, Clinton Yu, Lan Huang, Ping Xu, Peter Kaiser

**Affiliations:** 1 Department of Biological Chemistry, School of Medicine, University of California Irvine, Irvine, California, USA; 2 State Key Laboratory of Proteomics, Beijing Proteome Research Center, National Center for Protein Sciences (Beijing), Institute of Lifeomics, Beijing, China; 3 Department of Physiology & Biophysics, University of California Irvine, Irvine, California, USA

**Keywords:** Ubiquitin, K48, ubiquitin binding domain, ubiquitin interacting motif, met4, ubiquitin probe, 4xUb, tetra-ubiquitin, ACN, acetonitrile, BLI, biolayer interferometry, BSA, bovine serum albumin, DTT, dithiothreitol, DUB, deubiquitinating enzymes, E1, ubiquitin-activating enzyme, E2, ubiquitin-conjugating enzyme, E3, ubiquitin ligase, EDTA, ethylenediaminetetraacetic acid, FA, formic acid, IPTG, isopropyl β-d-1-thiogalactopyranoside, LC MS/MS, liquid chromatography tandem mass spectrometry, MGF, Mascot Generic Format, NHS, N-hydroxysuccinimide, PBS, phosphate-buffered saline, PMSF, phenylmethylsulfonyl fluoride, SILAC, stable isotope labeling by amino acids in cell culture, SRM, selective reaction monitoring, TBS, tris-buffered saline, tUBD, tandem ubiquitin binding domain, TUBEs, tandem ubiquitin binding entities, UBD, ubiquitin-binding domains, UIM, ubiquitin interacting motif, UIML, ubiquitin interacting motif like domain, XIC, extracted ion chromatogram, YPD, yeast extract peptone dextrose

## Abstract

Protein ubiquitylation is an important posttranslational modification that governs most cellular processes. Signaling functions of ubiquitylation are very diverse and involve proteolytic as well as nonproteolytic events, such as localization, regulation of protein interactions, and control of protein activity. The intricacy of ubiquitin signaling is further complicated by several different polyubiquitin chain types that are likely recognized and interpreted by different protein readers. For example, K48-linked ubiquitin chains represent the most abundant chain topology and are the canonical degradation signals, but have been implicated in degradation-independent functions as well, likely requiring a variety of protein readers. Ubiquitin binding domains that interact with polyubiquitin chains are likely at the center of ubiquitin signal recognition and transmission, but their structure and selectivity are largely unexplored. Here we report identification and characterization of the ubiquitin interacting motif-like (UIML) domain of the yeast transcription factor Met4 as a strictly K48-polyubiquitin specific binding unit using methods such as biolayer interferometry (BLI), pull-down assays, and mass spectrometry. We further used the selective binding property to develop an affinity probe for purification of proteins modified with K48-linked polyubiquitin chains. The affinity probe has a *K*_*d*_ = 100 nM for K48 tetra-ubiquitin and shows no detectable interaction with either monoubiquitin or any other polyubiquitin chain configuration. Our results define a short strictly K48-linkage-dependent binding motif and present a new affinity reagent for the K48-polyubiquitin-modified proteome. Our findings benefit the ubiquitin field in analyses of the role of K48-linked polyubiquitylation and increase our understanding of chain topology selective ubiquitin chain recognition.

## Highlights

• The UIML domain of Met4 is a K48-selective polyubiquitin binding domain.
• Development of a K48 polyubiquitin chain-specific probe using the UIML domain.
• Binding constants to K48 tetra-ubiquitin were determined by biolayer interferometry.
• Tandem arrangement of UIML increases affinity and maintains K48-chain specificity.
• The UIMLx2 probe can enrich K48 ubiquitylated proteins from yeast and HeLa cells.

## Graphical Abstract

**Figure:**
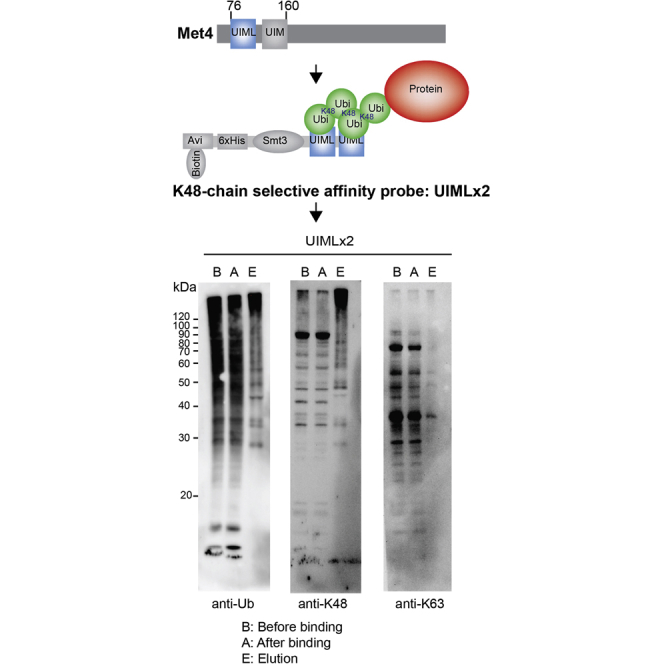

## In Brief

Ubiquitylation is a highly complex protein modification. The diversity of the ubiquitin signal is due to the variety of different ubiquitin chains that are assembled in cells. Villamil and colleagues identified a ubiquitin binding domain with high selectivity for only one specific chain type, K48-linked polyubiquitin. They used this UIML domain to generate a K48-ubiquitin chain selective affinity reagent for proteome-wide studies of proteins modified by this specific ubiquitin chain.

Ubiquitylation is a posttranslational modification that covalently attaches the small 76 residue ubiquitin protein to a lysine residue on target substrates. This covalent attachment is catalyzed by an enzymatic cascade that utilizes ubiquitin-activating enzyme (E1), ubiquitin-conjugating enzyme (E2), and ubiquitin ligase (E3) (1, 2). Ubiquitin itself can be further ubiquitylated through its seven Lys residues and the N-terminal Met (M1, K6, K11, K27, K29, K33, K48, and K63), leading to the formation of polyubiquitin and branched ubiquitin chains (3). Ubiquitylation is a reversible process where the ubiquitin moiety can be removed from the target substrates by deubiquitylating enzymes (4). Ubiquitin plays an important role in many signaling pathways and cellular functions such as protein degradation, signal transduction, cell cycle progression, immune response, and DNA damage repair (5, 6, 7, 8). Disruption within the ubiquitin signaling has been liked to various diseases including neurodegeneration, immune disorders, and cancer (9, 10, 11, 12).

Studies in the ubiquitin field have elucidated that monoubiquitylation is involved in DNA repair and endocytosis (13, 14). The different polyubiquitin chains can signal specific cellular responses, but many of them have been implicated in triggering proteasome-mediated degradation of substrates (15, 16). The most abundant and best-studied ubiquitin chain type is the K48-linked chain, which is responsible for degradation of the majority of proteasome substrates (17). K11 chains have also been associated in protein degradation, but are more prominent in targeting mitotic proteins for degradation during cell-cycle progression, as well as substrates of the endoplasmic-reticulum-associated degradation pathway (16, 18, 19). K63-linked and N-terminal (M1) linear polyubiquitin chains may be the only chain type with predominantly nondegradative roles. K63 chains have been associated with DNA repair (5, 20), and M1 chains have regulatory functions in the NF-κB signaling pathway (21). Most chain types are likely to confer different signaling functions depending on the substrate context. A well-studied example is the yeast transcription factor Met4, which is ubiquitylated by a homogeneous K48 chain, but not degraded by the proteasome (22, 23, 24). In the context of Met4, the K48 ubiquitin chain serves as an activity switch by repressing transactivation activity of the transcription factor.

Proteins can identify ubiquitin through specialized ubiquitin-binding domains (UBD), these ubiquitin “readers” are usually small segments within the protein that can identify ubiquitin and different ubiquitin moieties through noncovalent interactions (25, 26, 27). Many UBDs bind ubiquitin through a hydrophobic patch around I44 (L8, I44, H68, and V70). Besides the I44 hydrophobic patch, UBDs can also bind to an acidic hydrophilic surface around the D58, as well as the C-terminal end of ubiquitin (28). To date there are over 20 different families of UBDs that can interact with ubiquitin (29). A few UBDs were proposed to have some selectivity over other types of ubiquitin linkages. For example, the UBD of hHR23A (UBA2) has some preference to K48-linked ubiquitin chains, whereas the UBD of A20 (ZnF4) selectively binds to K63-linked ubiquitin chains and the UBAN domain recognizes linear (M1)-ubiquitin chains (30, 31, 32, 33). This binding selectivity is important for proper function of cellular processes. UBDs have binding affinities to ubiquitin that can range from low μM to mM levels. Many UBDs bind to monoubiquitin through weak interactions that can range from 10 to 500 μM. To overcome these, weak binding interaction proteins will have tandem UBDs than can help increase the binding affinity (1–15 μM) to polyubiquitin chains (28).

UBDs have been exploited to help in the identification of ubiquitylated proteins. One of the first studied ubiquitin-binding protein, S5a/Rpn10, was also used to help in the enrichment of ubiquitinated proteins (34). This enrichment of ubiquitin modified proteins by UBDs eventually led researchers to develop tandem ubiquitin-binding entities (TUBEs), which was an arrangement of several UBD sequences engineered into the same amino acid chain (35). These TUBEs were observed to have 100- to 1000-fold enhancement in K_d_ over a single UBD, they were used to help capture and purify all proteins that were modified by ubiquitin (28). TUBEs along with mass spectrometry helped in the identification of the ubiquitylated proteome in several organisms (35, 36, 37).

In this report we characterize the tandem UBD of the transcription factor Met4 and find a strict K48 linkage dependence for polyubiquitin binding for the UIML domain. We used the ubiquitin chain selective part of the Met4 tandem UBD to engineer a K48 chain selective ubiquitin affinity reagent, which can be used to selectively enrich proteins specifically modified with a K48-polyubiquitin chain.

## Experimental Procedures

### Cloning and Protein Purification

Yeast Met4 tUBD (amino acids 76–160), UIML (76–113), and UIM (114–160) were subcloned by PCR into a modified pET28(a) vector with an N-terminal Avi-6xHis-Smt3 tag using SalI/XhoI cut sites. Gibson assembly was used to create UIML repeats (UIMLx2, UIMLx3, and UIMLx4) (The plasmid for UIMLx2 is available from Adgene #174888). Three glycine residues were added as a linker in between each UIML domain. For protein production, BL21(DE3) cells were cotransformed with the Met4 UBD constructs and a pBirAcm plasmid encoding BirA for biotinylation of the Avi tag. The cells were grown in LB media at 37 °C with antibiotics kanamycin and chloramphenicol with the addition of 5 μg/ml biotin, to an OD_600nm_ ∼0.4, the temperature was reduced to 16 °C and expression was induced with 0.5 mM IPTG overnight. Cells were harvested and resuspended in lysis buffer (50 mM Tris, 500 mM NaCl, 2 mM PMSF, 10 mM imidazole, and 0.1% Triton X-100, pH 8.0), sonicated on ice, and the lysate was clarified by centrifugation at 4 °C for 20 min. The supernatant was incubated with Ni-NTA resin (GE Life science) for 1 h at 4 °C with constant tumbling before loading the Ni-NTA resin on a gravity column, where it was washed with 50 mM Tris (pH 8.0), 500 mM NaCl, and 20 mM imidazole. Recombinant proteins were eluted with 50 mM Tris (pH 8.0), 500 mM NaCl, and 250 mM imidazole. Protein samples were separated on a 12% SDS-PAGE and transferred to Immobilon-NC Transfer Membrane (Millipore), amido black staining was used to determine purity, and streptavidin-HRP was used to detect biotin incorporation. Pure fractions that contain biotin were collected and combined.

### Biolayer Interferometry

Binding preference and affinity of tetra-ubiquitin chains (M1, K6, K11, K29, K33, K48 and K63, Boston Biochem) were determined on a ForteBio Octet RED96 (Pall ForteBio LLC). Protein samples were diluted in assay buffer (50 mM Tris, 150 mM NaCl, 0.01 mg/ml BSA, and 0.02% tween 20, pH 7.5) as follows: Met4 UBDs final concentration 20 μg/ml, biocytin (quenching solution) 5 μg/ml, and the tetra-ubiquitin chains were diluted in a range between 1.45 and 0.0028 μM. Streptavidin Dip and Read biosensors (ForteBio) were hydrated with the assay buffer prior to the experiment. The monitoring conditions were as follows: initial baseline for 60 s, loading for 120 s, quenching for 120 s, baseline for 30 s, association for 30 s, and dissociation for 90 s; shake speed 1000 rpm, and plate temperature remained at a constant 30 °C. To examine the binding constants, the data were analyzed using ForteBio Data Analysis 9.0. To determine binding preference to the tetra-ubiquitin chains (M1, K6, K11, K29, K33, K48, and K63), a final concentration of 0.15 μM tetra-Ub was used. To calculate the binding constants, the following concentration of K48 tetra-ubiquitin chain was used: 1450, 483, 161, 53, 17, 5.6, and 2.8 nM.

### Biotin-Protein Immobilization for K48 PolyUb Pull-Down in Mammalian Cells

Purified Biotin-Met4(UIMLx2) was incubated with streptavidin sepharose affinity resin in TBS at 4 °C for 1 h with gentle rocking. After incubation the unbound fraction was removed and the resin washed twice with 1 ml TBS. The resin was then treated with 250 μl of biotin blocking solution (TBS with 40 μM biotin) for 5 min at room temperature before the biotin blocking solution was removed and the resin washed twice with 1 ml TBS.

### Cell Culture and PolyUb Pull-Down Assay

Five 150 mm dishes of HeLa cells were grown to 80% confluency and treated with 10 μM MG132 for 1 h before harvesting. The cells were briefly washed with PBS and then resuspended in lysis buffer (50 mM Tris pH 7.5, 150 mM NaCl, 1 mM EDTA, 0.5% NP40) supplemented with protease inhibitors (1 mM PMSF, 2 μM pepstatin, 10 μM leupeptin) and DUB inhibitor (1 mM iodoacetamide and 8 mM 1,10-o-phenanthroline). Cells were sonicated on ice and the protein extracts were then centrifuged at 100,000*g* for 20 min at 4 °C. The supernatant was removed and incubated with streptavidin sepharose affinity resin that contains the biotinylated UIMLx2 bait at 4 °C for 1 h with gentle mixing. After incubation the unbound fraction was removed and the resin was washed twice with 1 ml of cold lysis buffer. Bound protein samples were eluted with 8 M urea. Samples were separated on a 12% SDS-PAGE and transferred to a membrane and further analyzed with either anti-Ub (Santa Cruz), anti-K48 (Cell Signaling), or anti-K63 (Cell Signaling) antibody.

### PolyUb Pull-Down Assay With High Salt and Urea Wash

HeLa cells were grown, treated with MG132, and lysed as above. The supernatant was incubated with streptavidin sepharose affinity resin bound with biotinylated UIMLx2 bait as before and the unbound samples were removed. The resin was washed twice with 1 ml of wash buffer with increasing NaCl concentration (50 mM Tris pH 7.5, [150, 250, 500, or 1000 mM] NaCl) or a wash buffer with increasing Urea concentration (50 mM Tris pH 7.5, 150 mM NaCl, [0, 0.5, 2, 4, or 8 M] Urea). Bound protein samples were eluted from all samples with 8 M urea and separated by SDS-PAGE. The samples were transferred to a membrane and analyzed using anti-K48 antibodies.

### Sample Preparation for Mass Spectrometry (Mammalian Cells)

The eluted protein samples were reduced with 5 mM DTT for 30 min at 37 °C. Iodoacetamide was added to a final concentration of 10 mM for 30 min at room temperature in the dark. After iodoacetamide treatment, 5 mM DTT was added for 10 min at room temperature. Samples were digested with LysC (Promega) at 37 °C for 4 h. After LysC digestion, the urea concentration was reduced to 1.5 M using 100 mM NH_4_HCO_3_, and trypsin was added to the sample for overnight digestion at 37 °C. The digested samples were desalted and concentrated using C18 zip tip.

### Mass Spectrometric Analysis of Mammalian Samples

The resulting peptides were subjected to LC MS/MS using an UltiMate 3000 RSLC (Thermo Fisher) coupled to an Orbitrap Fusion Lumos mass spectrometer (Thermo Fisher). LC analysis was performed on a 25 cm × 75 μm I.D. Acclaim PepMap RSLC column. Peptides were eluted using a gradient of 3% to 25% B in 60 min at a flow rate of 300 nl/min (solvent A: 100% H_2_O, 0.1% formic acid; solvent B: 100% acetonitrile, 0.1% formic acid). Raw spectrometric files were converted to MGF using MSconvert (ProteoWizard 3.0.10738) and were searched using Batch-Tag within a developmental version (v. 6.0.0) of Protein Prospector at the University of California, San Francisco, against a decoy-containing database consisting of a normal homo sapiens Swissprot database concatenated with a randomized version (2019.04.08; 20,418 entries). The mass accuracy for parent ions and fragment ions were set as ±20 ppm and 0.6 Da, respectively. Trypsin was set as the enzyme, and a maximum of two missed cleavages were allowed. Protein N-terminal acetylation, methionine oxidation, N-terminal conversion of glutamine to pyroglutamic acid, and GlyGly modifications on lysine residues were selected as variable modifications, while cysteine carbamidomethylation was set as a fixed modification. The minimum score and maximum expectation value for each individual spectrum were set to 15 and 0.05, respectively. Maximum FDR for proteins and peptides were both set to 1% using a target-decoy approach.

### UIMLx2 Domain Immobilization for Yeast Experiments

The purified UIMLx2 domain was eluted and then resuspended in coupling buffer (0.2 M NaHCO_3_, 0.5 M NaCl pH 8.3) before being coupled to NHS-activated Sepharose (GE Healthcare) following the manufacturer’s instructions. The UIML2-conjugated agarose was stored at 4 °C in PBS supplemented with 30% glycine (36).

### Affinity Purification of UIMLx2 Interacting Yeast Proteins

The yeast strain JMP024 (38) was grown at 30 °C in yeast extract peptone dextrose (YPD) medium (1% yeast extract, 2% Bacto-peptone, 2% dextrose), and harvested in the early log phase (OD_600nm_ = 1.0). Cells were then resuspended in lysis buffer (50 mM Na_2_HPO_4_ pH 8.0, 500 mM NaCl, 0.01% SDS, 5% glycerol, 5 mM iodoacetamide) and broken with glass beads. Protein extracts were centrifuged at 70,000*g* for 30 min and then incubated with immobilized UIML2 beads at 4 °C for 30 min. After incubation, the beads were washed with lysis buffer and then wash buffer (50 mM Na_2_HPO_4_ pH 8.0, 5 mM iodoacetamide), followed by 50 mM Na_2_HPO_4_ pH 8.0 to remove iodoacetamide. Bound proteins were eluted by boiling in 1× SDS-PAGE loading buffer (50 mM Tris-HCl pH 6.8, 2% SDS, 10% glycerol, 0.1% bromophenol blue, 1% β-mercaptoethanol).

### Targeted Quantitative MS Analysis of UIMLx2 Purified Yeast Polyubiquitin Linkages Using SILAC-Based Approach

To accurately quantify the abundance of the seven polyubiquitin chains from ubiquitin conjugates purified with the UIMLx2, heavy isotope-labeled ubiquitin conjugates (K6, R10) were purified with Ni-NTA beads as a spike quantification standard for the UIMLx2 purified ubiquitin conjugates, Tier level of our SRM experiment is Tier 2.

Briefly, the JMP024 yeast strain was cultured approximately eight generations to reach OD_600nm_ of 0.7 in synthetic media (0.7% Difco yeast nitrogen base, 2% dextrose, supplemented with adenine, uracil, and amino acids). The synthetic media was also supplemented with 12 mg/l of [^13^C_6_^15^N_4_] arginine (+10.0083 Da) and 18 mg/l [^13^C_6_] lysine (+6.0201 Da). The labeled cells were broken with the glass beads beating method, then subjected to Ni-NTA purification. Afterward, the Ni-NTA-purified ubiquitin conjugates were mixed with the UIMLx2 purified ubiquitin conjugates, then digested by trypsin prior to LC-MS/MS analysis.

The digested peptides were dissolved in a sample buffer (1% FA, 1% ACN), loaded on an Easy 1200 nano LC system, separated on an in-house packed capillary column (ID 75 μm × 15 cm) with 3-μm C18 reverse-phase fused silica (Michrom Bioresources, Inc), and eluted by a linear gradient from 4 to 35% of mobile phase B (phase B, 0.1% FA in 98% ACN; phase A, 0.1% FA, 2% ACN in water) at a flow rate of 0.3 μl/min. The eluted peptides were analyzed by a hybrid LTQ-Orbitrap Velos mass spectrometer (MS) (Thermo Fisher Scientific) sequentially. Eluted peptides were detected on an Orbitrap mass spectrometer in a survey scan (300–1600 m/z, resolution 30,000) followed by selective reaction monitoring (SRM) scans for three ubiquitin chains ions in the LTQ. The peptide intensity for quantification was manually analyzed by ion chromatograms (XIC) using Xcalibur v3.0 software (Thermo Finnigan). The raw data files were transformed into MGF file using MSconvert in Proteowizard 3.0.10158, then searched against a human proteome database (20,380 entries, February 27, 2018) downloaded from uniprot (https://www.uniprot.org/). A fixed modification of carbamidomethylation on cysteine (+57.0215 Da) and variable modifications of oxidation on methionine (+15.9949 Da) and di-glycine on lysine (+114.0429 Da) were used. Individual spectra from Maxquant with score >40, modification localization probability >0.75 were accepted as confidently identified. For global identification, a false discovery rate (FDR) was calculated base in a target-decoy mode.

### Experimental Design and Statistical Rationale

For the mammalian cell experiment, a total of four samples were analyzed, two UIMLx2 and two Smt3 biological replicates were used. Both sets of samples were treated in the same manner as described above for capturing mammalian cell proteins and mass spectrometry analysis. The protein samples pulled down by Smt3 was used as the control for the protein samples from UIMLx2. The UIMLx2 probe has a Smt3 tag at the N-terminal end to help with protein stability and as a spacer between the biotin tag and the UIMLx2 probe.

### Deubiquitylation Protection Assay

The deubiquitylating enzyme Otubain-1 or USP2 (BostonBiochem) was incubated first in the assay buffer (50 mM Tris pH 7.5, 150 mM NaCl, 5 mM DTT, 0.1 mg/ml BSA) for 10 min at room temperature. K48 tetra-ubiquitin at a concentration of 1.3 μM was incubated with either 5.2 μM UIMLx2, 5.2 μM SMT3 or nothing in assay buffer for 10 min at 37 °C. In total, 1.5 μM Otubain-1 or 10 nM USP2 was added to the mixture, incubated at 37 °C, and samples were taken at time point 0, 30, 60, and 120 min. The reaction was quenched in loading dye and heated to 100 °C for 10 min. The samples were separated by SDS-PAGE and then transferred to a membrane for analysis with anti-ub antibodies.

## Results

### Characterization of Binding Properties of the Met4 Tandem Ubiquitin Binding Domain

**Fig. 1:**
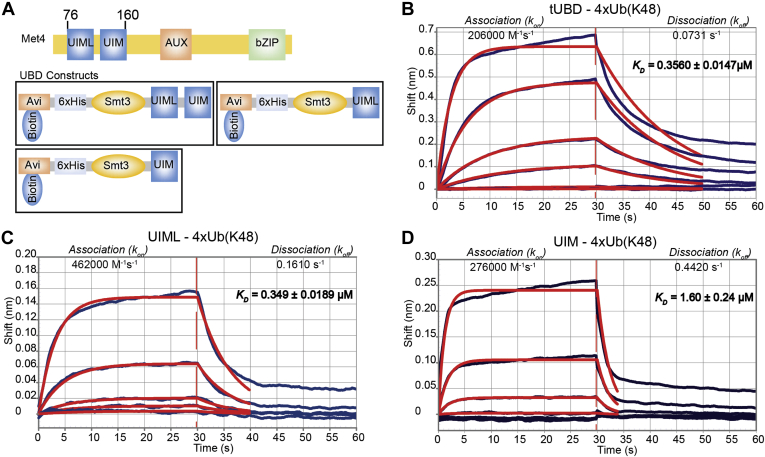
**The Met4 tUBD binds to K48tetra-ubiquitin.***A*, Met4 functional domains (*top*), and the three different constructs of the tUBD, UIML, and UIM domains. These constructs have an N-terminal biotinylation site, 6xHis tag, and a Smt3 tag. *B*–*D*, binding graphs displaying the association and dissociation of K48 tetra-ubiquitin from Met4 residues 76 to 160 (tUBD) (*B*), Met4 residues 6 to 113 (UIML) (*C*), and Met4 residues 114 to 160 (UIM) (*D*). Biolayer interferometry was used to obtain binding data.

Met4 is a yeast transcription factor that contains two ubiquitin binding domains in close proximity, which is referred to as tandem UBD (tUBD) (Fig. 1*A*). One of the UBDs is a ubiquitin interacting motif (UIM) (23), whereas the other UBD is a ubiquitin interacting motif-like domain (UIML) (39). To characterize affinity and selectivity of the tUBD of Met4, we generated recombinant biotinylated fragments of Met4 spanning residues 76 to 160 (tUBD), residues 76 to 113 (UIML), and residues 114 to 160 (UIM) (Fig. 1*A* and supplemental Fig. S1). We have previously demonstrated by pull-down assays that the tUBD binds K48 ubiquitin chains starting at a chain length of three ubiquitins (39) and therefore used K48-linked tetra-ubiquitin (4xUb(K48)) to characterize binding parameters of the tUBD and the individual UIM and UIML domains, respectively (Fig. 1). We used biolayer interferometry (BLI) (40) for these experiments with biotinylated purified UBDs attached to a streptavidin sensor. Consistent with previous findings (41), the binding constant of 4xUb(K48) to the Met4 tUBD was about 350 nM (Fig. 1*B* and Table 1). Interestingly, the isolated UIML domain showed about the same dissociation constant as the tUBD (Fig. 1*C* and Table 1), whereas the UIM domain has a comparably lower K_D_ of 1.6 μM, which is typical for UIM domains (Fig. 1*D* and Table 1). The UIM does therefore not contribute to the overall binding constant of the tUBD of Met4. However, the UIM domain has clearly an important function *in vivo* because blocking the ubiquitin binding activity of the UIM with single point mutations reduces protection of ubiquitylated Met4 from proteasomal degradation (23, 39). Inspection of kinetic parameters of ubiquitin binding can explain this important function of the UIM domain (Table 1) as its presence in the tUBD reduces the dissociation rate for 4xUb(K48) and thus results in a less dynamic interaction.

**Table 1:** Met4 UBD binding constants to K48 tetra-ubiquitin

Met4 UBD	On-rate *k*_*on*_ (M^−1^s^−1^)	Error *k*_*on*_ (M^−1^s^−1^)	Off-rate *k*_*off*_ (s^−1^)	Error *k*_*off*_ (s^−1^)	Dissociation constant *K*_*D*_ (μM)	Error *K*_*D*_ (μM)
tUBD (76–160)	206,000	7920	0.0731	0.00111	0.356	0.0147
UIML (76–113)	462,000	23,900	0.161	0.00263	0.349	0.0189
UIM (114–160)	276,000	38,800	0.442	0.0212	1.60	0.24

### The Met4 UIML Domain Binds Specifically to K48 Linked Tetra-Ubiquitin

**Fig. 2:**
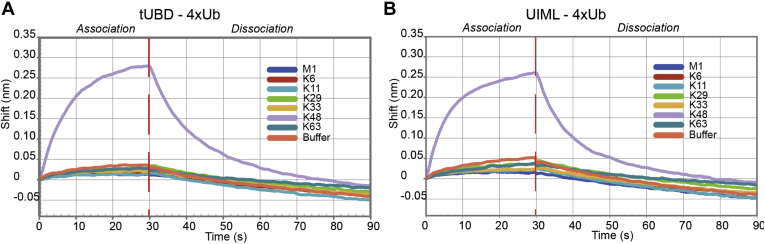
**K48 chain selective binding.** Met4 tUBD (*A*) and UIML (*B*) binding to tetra-ubiquitin (M1, K6, K11, K29, K33, K48, and K63) was determined using biolayer interferometry. K48 tetra-ubiquitin selectively binds to tUBD (*A*) and UIML (*B*).

Most UBDs bind various ubiquitin chain types. We therefore tested the binding of the tUBD with commercially available tetra-ubiquitin chains linked through different lysine residues (M1, K6, K11, K29, K33, K48, and K63) using BLI. All naturally occurring homotypic ubiquitin chains, but the K27-linked ubiquitin chain, which is not commercially available, were tested for binding to the Met4 tUBD. Surprisingly, only the 4xUb(K48) displayed any detectable binding to the tUBD construct (Fig. 2*A*). As previously demonstrated no binding was observed for K11 chains, which together with K48 chains is the physiological chain attached to Met4 on a lysine 163 (41). This surprisingly selective recognition of only K48 linked ubiquitin chains is a unique characteristic of the Met4 tUBD as other ubiquitin binding domains typically display promiscuous binding to various ubiquitin chain structures (42, 43).

Ubiquitin binding of the tUBD is driven by the UIML domain, whereas the UIM region affects kinetic parameters of complex formation (Fig. 1 and Table 1). We therefore tested whether the UIML displays intrinsic chain selectivity, or if the combination of UIML and UIM determines ubiquitin chain topology selective interaction. The isolated UIML showed the same K48 chain selectivity as the tUBD (Fig. 2*B*), demonstrating that the UIML domain dictates not only binding affinity, but also chain topology-specific interaction of the tUBD of Met4.

### Tandem UIML Constructs Increase Binding to K48 Tetra-Ubiquitin Chains

**Fig. 3:**
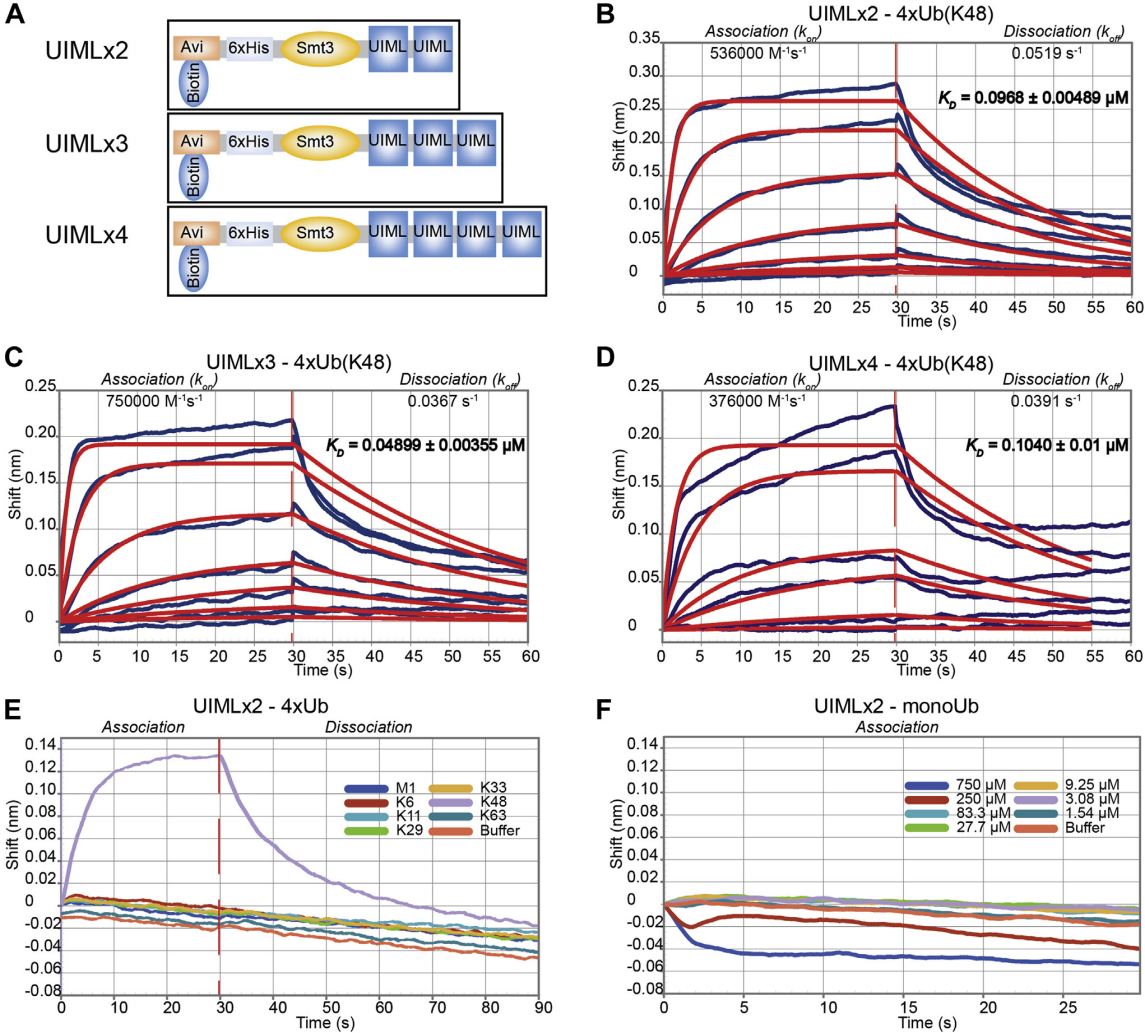
**Tandem UIML binding to K48 tetra-ubiquitin.***A*, UIML probe constructs with repeats of Met4 residues 76 to 113. All probes contain the Avi-6xHis-Smt3 tag. *B*–*D*, binding data displaying association and dissociation of K48 tetra-ubiquitin to UIMLx2 (*B*), UIMLx3 (*C*), and UIMLx4 (*D*). *E*, UIMLx2 selective binding to K48 tetra-ubiquitin in comparison to other tetra-ubiquitin topologies (M1, K6, K11, K29, K33, and K63). *F*, UIMLx2 binding to increasing concentrations of monoubiquitin.

The strict K48 selectivity of ubiquitin binding of the UIML domain prompted us to explore development of an ubiquitin binding probe. Three constructs were created in which UIML domain (76–113) was repeated 2, 3, and 4 times in tandem with a three amino acid linker sequence separating the individual domains. All constructs had an Smt3 tag and could be biotinylated *in vivo* based on the Avi tag (Fig. 3*A* and supplemental Fig. S2). Proteins were purified and ubiquitin binding was characterized by BLI (Fig. 3, *B*–*D* and Table 2). Two copies of the UIML (UIMLx2) resulted in a markedly improved dissociation constant (97 nM), considering that ubiquitin binding is typically in the μM to mM range. All of the new tandem constructs (UIMLx2, UIMLx3, and UIMLx4) displayed lower *K*_D_ values indicating tighter binding to K48 tetra-ubiquitin chains compared with just UIML (76–113) (Fig. 3, *B*–*D* and Table 2). Extending repeat numbers beyond UIMLx2 only slightly improved binding parameters, we decided to proceed with UIMLx2 because it displayed better stability and expression compared with UIMLx3 and UIMLx4.

**Table 2:** UIML binding constants to K48 tetra-ubiquitin

UIML construct	On-rate *k*_*on*_ (M^−1^s^−1^)	Error *k*_*on*_ (M^−1^s^−1^)	Off-rate *k*_*off*_ (s^−1^)	Error *k*_*off*_ (s^−1^)	Dissociation constant *K*_*D*_ (μM)	Error *K*_*D*_ (μM)
UIMLx2	536,000	25,500	0.0519	0.000858	0.0968	0.00489
UIMLx3	750,000	52,000	0.0367	0.00076	0.0489	0.00355
UIMLx4	376,000	27,400	0.0391	0.00137	0.104	0.01

We next tested whether UIMLx2 retained the same ubiquitin chain selectivity as UIML (76–113). As previously we used commercially available tetra-ubiquitin (M1, K6, K11, K29, K33, K48, and K63) to measure binding to UIMLx2 (Fig. 3*E*). UIMLx2 retained its complete selectivity toward K48 tetra-ubiquitin (Fig. 3*E*). In addition, we tested if the UIMLx2 bound to free mono-ubiquitin. Using a concentration between 1.54 μM and 750 μM, we found no binding between UIMLx2 and free monoubiquitin (Fig. 3*F*). Thus, the UIMLx2 probe is highly selective for K48 ubiquitin chains and does not interact with monoubiquitin.

### K48 Chain Selective Purification of Ubiquitylated Proteins From Yeast

**Fig. 4:**
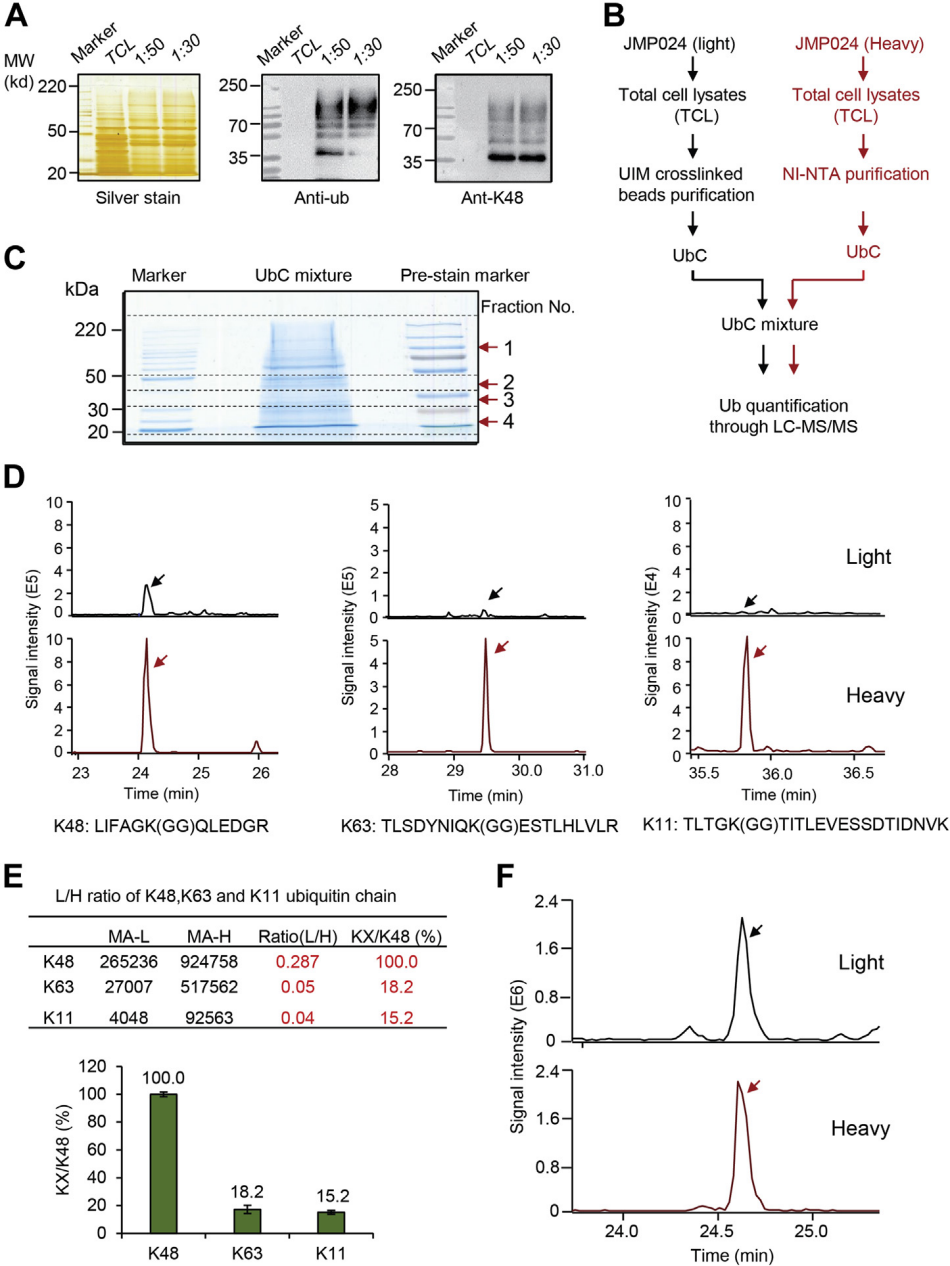
**UIMLx2 selectively binds to K48 ubiquitylated proteins in yeast cell lysates.***A*, UIMLx2 was incubated with yeast cell lysates at two different ratios (1:50 and 1:30) and bound proteins were analyzed by immunoblotting with anti-ubiquitin or anti-K48 antibodies. *B*, diagram of SILAC experiment. Yeast strain JMP024, which expresses 6xHis tagged ubiquitin was cultured with either light or heavy isotopes. Cells were lysed and bound to UIMLx2 cross-linked to beads or Ni-NTA for unbiased ubiquitin pull down. Proteins conjugates with ubiquitin (UbC) were eluted, mixed, and separated by SDS-PAGE. Gel slices were then processed for MS analyses. *C*, gel of separated ubiquitylated proteins were separated into four segments. *D*, mass spectrometry analysis of gel segment one showing peptide peaks corresponding to K48 (LIFAGK(GG)QLEDGR), K63 (TLSDYNIQK(GG)ESTLHLVLR), and K11 linkages (TLTGK(GG)TITLEVESSDTIDNVK). *E*, ratio of light/heavy K48, K63, and K11 ubiquitin chains of gel segment one. The ratio for K48 was designated as 100%, K63 linkage encompasses 18.2% of the total K48 linkages, and K11 encompasses 15.2% (coefficient of variation was below 15% between replicates). *F*, mass spectrometric analysis of gel fraction 3 indicating mostly K48-linked ubiquitin.

This work so far suggests that the UIMLx2 specifically binds to purified K48 tetra-ubiquitin *in vitro*. We next wanted to determine if this probe can be used to capture proteins modified with K48 ubiquitin chains from cell lysates. The UIMLx2 probe was covalently bounded to NHS beads and then incubated with total yeast cell lysates at two different ratios (UIMLx2: cell lysate, 1:50 and 1:30). Beads were washed and bound proteins eluted for analyses. Ubiquitylated proteins were efficiently purified from total protein lysates (Fig. 4*A*).

Next, we wanted to determine the types of ubiquitin conjugates that are bound to the UIMLx2 probe. To this end we utilized yeast cells expressing polyhistidine tagged ubiquitin and used SILAC to quantitatively compare ubiquitin chain topologies purified on UIMLx2 or Ni-NTA (total cellular ubiquitin) (Fig. 4*B*). Both purifications were mixed and separated by SDS-PAGE and the gel was sectioned into four parts (Fig. 4*C*). Fraction 1 (proteins >50 kDa) and fraction 3 had a strong K48 signal, whereas fractions 2 and 4 had little to no K48 ubiquitin chain signal (Fig. 4, *A* and *C*). To evaluate ubiquitin chain topology selective purification, signature peptides for the most abundant chain types (K48, K63, and K11) were analyzed by detecting the following peptides with “GG-remnants” corresponding to ubiquitin chain K48 (LIFAGK(GG)QLEDGR), K63 (TLSDYNIQK(GG)ESTLHLVLR), and K11 (TLTGK(GG)TITLEVESSDTIDNVK) (Fig. 4*D*, supplemental Fig. S5 and supplemental Table S1) (44). No other linkages were detected in our samples. The ratio of light to heavy of the same peptide fragment was calculated and normalized to be 100% for the K48 signature peptide. Analyses of gel fragment 1, which contains high molecular conjugates, showed that most of the K63 and K11 chains were lost during purification on UIMLx2 affinity resins (Fig. 4*E*). Given the lack of any detectable affinity of UIMLx2 for K63 or K11 chains (Fig. 3*E*), the detected low levels of K63 and K11 linkages are likely a result of copurified proteins with K11 or K63 chains, proteins modified with mixed chains, or proteins modified with two different ubiquitin chain types. Gel fragment 3 contains lower complexity conjugates, and only K48 ubiquitin chain linkages were detected (Fig. 4*F* and supplemental Fig. S5). These results demonstrate selective purification of K48-linked ubiquitin chains from cell lysates.

### Detecting Proteins Modified With K48 Linked Ubiquitin Chains in HeLa Cells

**Fig. 5:**
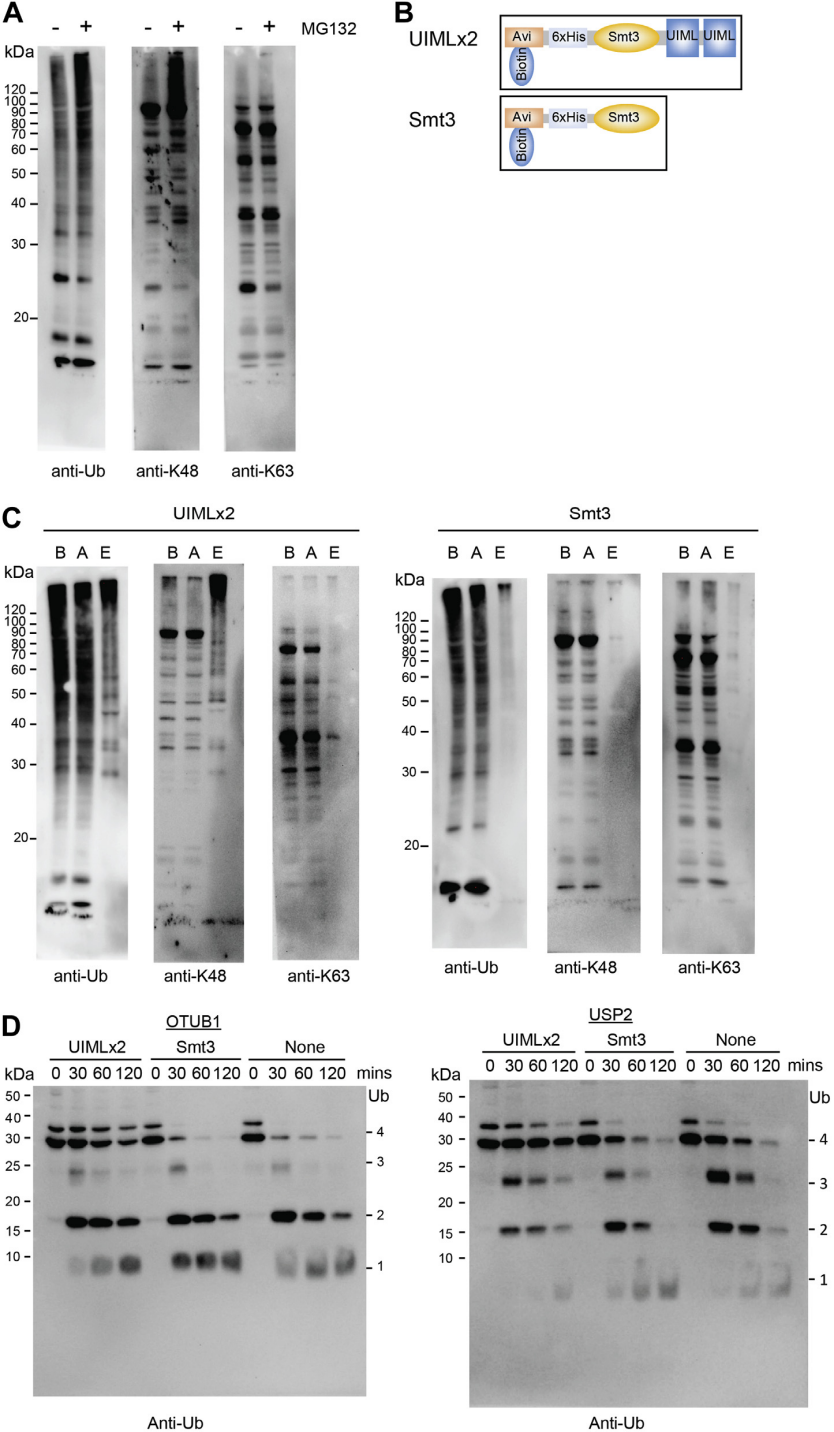
**UIMLx2 binds to K48 ubiquitylated proteins in HeLa cell lysates and hinders DUB cleavage.***A*, cell lysates were analyzed by immunoblotting with antibodies indicated. Proteasome inhibitor MG132 increases ubiquitylated proteins in cells. *B*, schematic of the UIMLx2 and control Smt3 probes used for affinity purification. *C*, samples purified from HeLa whole cell lysates using UIMLx2 or the Smt3 probe were separated by SDS-PAGE and analyzed with antibodies indicated. UIMLx2 displays an affinity for K48 linked ubiquitinated proteins in HeLa cells (“B”: clarified lysate sample before binding; “A”: clarified lysate sample after binding; “E”: elution of bound sample). *D*, UIMLx2 *in vitro* DUB protection. UIMLx2 hinders deubiquitylation of K48 tetra-ubiquitin from OTUB1 and USP2. Time points of the reaction were taken at 0, 30, 60, and 120 min, samples were quenched in loading dye and heated to 100 °C. Anti-Ub was used to detect the ubiquitin bands.

The UIMLx2 affinity resin can selectively purify K48 ubiquitin chains from yeast cells. We next sought to determine if UIMLx2 can be used to purify the K48-chain modified human proteome. K48-linked ubiquitin chains are the canonical degradation signal and modified proteins are thus typically rapidly degraded by the 26S proteasome (45). To increase the abundance of these degradation intermediates, HeLa cells were treated with the proteasome inhibitor MG132 before purification with UIMLx2 (Fig. 5*A*). The UIMLx2 was expressed and purified as an Avi-6xHis-Smt3 tagged UIMLx2 fusion protein, and we therefore used the Avi-6xHis-Smt3 fragment as our control (Fig. 5*B*). We first tested if the UIMLx2 discriminates between K48 and K63 ubiquitylated proteins in HeLa cell lysates by immuno blotting with K48 and K63 chain selective antibodies. Affinity purification was performed with the UIMLx2 probe and the Avi-6xHis-Smt3 fragment as a control (Fig. 5). The eluted fraction from the UIMLx2 probe is strongly enriched for K48-linked ubiquitin chains and almost no signal for K63-linked chains was detected (Fig. 5*C*). The control Avi-6xHis-Smt3 fragment did not purify significant amounts of polyubiquitylated proteins (Fig. 5*C*). To test binding properties between the UIMLx2 and K48-linked ubiquitin chains, we used stringent wash conditions with increasing NaCl or urea concentrations before elution with 8 M urea and detection of K48-polyubiquitin by immunoblotting with anti-K48-ubiquitin antibodies. HeLa cell lysates were used as a source of K48-ubiquitylated proteins. Binding was stable up to 250 mM NaCl, but significant loss of K48-ubiquitin chains was observed at 500 and 1000 mM NaCl (supplemental Fig. S3*A*). In contrast, the interaction between UIMLx2 and K48-linked ubiquitin chains was very sensitive to urea, with significant loss of binding at 0.5 M urea indicating that hydrophobic interactions drive binding (supplemental Fig. S3*B*). Overall, these results are in accordance with the previous results using yeast cells and showed that the UMILx2 probe can enrich K48-linked polyubiquitylated proteins from mammalian cell extracts.

We next tested whether the UIMLx2 probe can function as a deubiquitylation protector, by restricting access of DUBs. K48 tetra-ubiquitin chains were incubated with UIMLx2, SMT3, or buffer for several minutes before adding the deubiquitylating enzyme. Two DUBs were used, the K48 ubiquitin chain specific OTUB1 and the less specific USP2 (catalytic domain) (46, 47, 48, 49), to cleave the K48 tetra-ubiquitin chains. OTUB1 was able to process the K48 tetra-ubiquitin chains after 30 min (Fig. 5*D*) and after 120 min a majority is cleaved to mono-ubiquitin. The Smt3 displayed no protection from OTUB1 activity as the cleavage pattern is similar to the control (Fig. 5*D*). UIMLx2 strongly delayed the cleavage of K48 tetra-ubiquitin by OTUB1 with K48 tetra-ubiquitin still detectable after 120 min (Fig. 5*D*). Similar results were obtained with USP2 (Fig. 5*D*). These results demonstrate that the UIMLx2 can protect K48 ubiquitin chains from DUB cleavage.

**Fig. 6:**
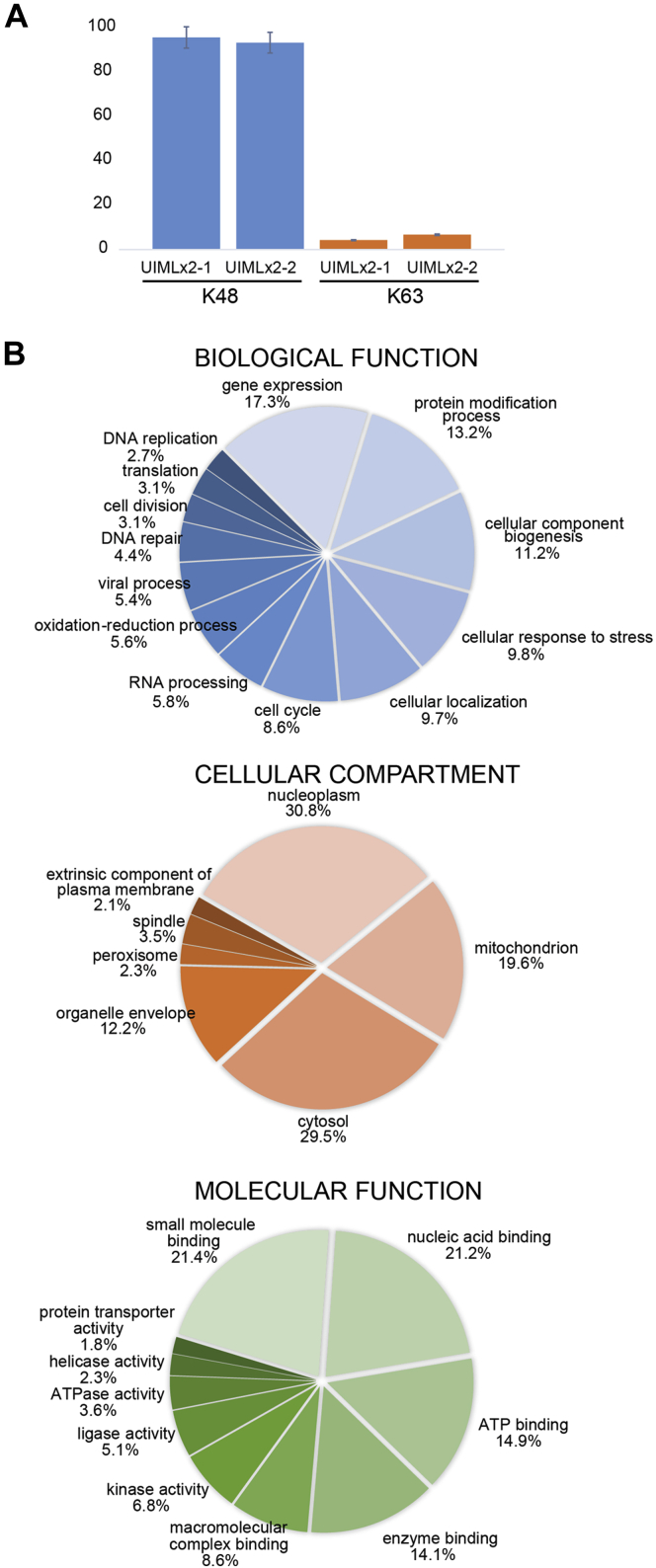
**Analysis of proteins isolated from HeLa cells.***A*, distribution of ubiquitin linkages isolated from HeLa cells. *B*, DAVID analysis using GO terms to separate biological function, cellular compartment, and molecular function.

We next aimed to identify human proteins that were enriched on the UIMLx2 affinity resin and thus likely modified with a K48-linked ubiquitin chain. Two independent experiments were performed and analyzed by LC-MS/MS. ProteinProspector was used for data analyses against the human proteome sequence obtained from UniProt. A protein identification list was created listing all the proteins that were eluted from the UIMLx2 probe and the Avi-6xHis-Smt3 control (supplemental Table S2). Only proteins that appeared in both duplicates for each probe were further analyzed. In total, 411 proteins were uniquely identified from the UIMLx2 probe. Ubiquitin linkage analyses using GG-remnant signature peptides in samples eluted from the UIMLx2 probe showed over 90% of the linkages were on K48 with only a small fraction on K63 (Fig. 6*A*). These results corroborate well with those revealed by immunoblotting analysis as described above (Fig. 5*C*). This is also in accordance with the results found in yeast cells where the UIMLx2 probe primarily bound to K48 ubiquitin chains (Fig. 4*E*).

Proteins specifically bound to the UIMLx2 probe were further analyzed to determine their function and localization. Analysis with DAVID (Database for Annotation, Visualization and Integrated Discovery) Bioinformatics Resource to identify enriched biological themes using Gene Ontology terms showed that proteins specifically bound to the UIMLx2 probe had a rage of biological functions including gene expression, protein modification, cellular component biogenesis, cellular response to stress, localization, cell cycle, and others (Fig. 6*B*). We next looked into cellular compartmentalization of the K48 ubiquitylated proteins and found that many of them are localized in the nucleoplasm, mitochondrion, and cytosol (Fig. 6*B*). It was interesting to see that one of the top three cellular localizations for K48 ubiquitylated proteins was the mitochondrion. Further classification of these proteins using the MitoCarta3.0 database (50) showed that 56% and 38% of the identified mitochondrial proteins with known localization where matrix or inner mitochondrial membrane proteins, respectively (supplemental Fig. S4). Identified proteins are thus likely substrates of quality control mechanisms at the mitochondria (51). Finally, we looked into the molecular functions of the proteins and found enrichment for binding small molecules, nucleic acids, and ATP (Fig. 6*C*).

## Discussion

Ubiquitylation was initially solely thought of as a degradation signal, but is now being recognized as one of the most complex signals controlling proteolytic and nonproteolytic pathways. The complexity stems from the diversity of the modification, with mono, multi, and polyubiquitylation forming the most basic forms. Polyubiquitylation comes in eight different homotypic chain types based on the seven lysines in ubiquitin and the amino-terminus used for chain formation (1, 12, 13, 14, 15, 16). Complexity is further increased by chain branching, mixed chain types, and incorporation of ubiquitin with posttranslational modifications (16, 17, 18, 19, 20, 21, 22, 23, 24). The complexity of ubiquitin signals requires sensors/readers for signal detection and processing. UBDs fulfill this function (32, 33). Ubiquitin chain-type selective UBDs are predicted to play an important role in interpretation of different signals mediated by the different chain types. Very few chain selective UBDs have been identified, and selectivity is often not absolute or requires bivalent interactions to achieve selectivity. We have shown here that the small UIML domain, first identified in the yeast transcription factor Met4 (39), has absolute specificity for K48-linked polyubiquitin chains, without detectable binding to other ubiquitin chains. This strict K48 chain selectivity is at the center of regulation of the transcription factor Met4, because the same domain binds the mediator complex (41). K48-chains compete with mediator binding and block transactivation, while other chains do not. Therefore an ubiquitin chain topology switch serves as an on/off switch for transcription factor activity (41).

The UIML domain is part of a tandem UBD in Met4. We found that the UIML was the primary binding component of tUBD and drives affinity and K48-chain selectivity. The role of the UIM region is clear from biological experiments, where it is essential for Met4 regulation (23, 39). While contribution to overall affinity and chain selectivity was not detected, we show that the combination of UIML and UIM reduces the dissociation rate and thus likely decreases the dynamic nature of the interaction.

Given the exceptional selectivity of the UIML domain for K48 ubiquitin chains, we develop a tandem UIML construct to be used as an affinity probe to selectively bind to K48 ubiquitylated proteins. The UIMLx2 probe maintained the strict selectivity for K48 ubiquitin chins with increased affinity. We tested the UIMLx2 probe in both yeast and mammalian cell extracts and demonstrate that UIMLx2 can selectively purify K48 ubiquitylated proteins from total cell lysates. In both lysates K48 chain selectivity was maintained with only small amounts of K63 and K11 chains detected in purified samples. Given the absence of any detectable binding to any other chain but K48 *in vitro*, we believe the detected K63 and K11 chains could indicate proteins with multiple chain types or mixed/branched chains.

Given the excellent selectivity and good affinity, the UIMLx2 probe can likely be further developed into an *in vivo* sensor to determine K48-ubiquitin chain function, localization, and dynamics as has been achieved for K63 ubiquitin chain sensors (52). It is also feasible to combine the UIMLx2 with other protein specific probes to help determine localization of specific K48 linked ubiquitylated proteins, as has been demonstrated by the Cohen group for detection of ubiquitylated nucleosomes in live cells (53).

How the UIML discriminates K48-linked chains from all other ubiquitin chain topologies will be an interesting question to address in future experiments. One can imagine two different modes of interactions, recognition of the K48 linkage region or simultaneous interaction with multiple ubiquitins such that binding surfaces are only exposed in the correct topology when ubiquitins are linked through K48. Our experiments cannot distinguish between these two interaction modes, but the relatively high affinity suggests that relatively large interaction surfaces are engaged.

In summary, the UIMLx2 probe with its strict K48-linkage dependence we report here can be a powerful tool to selectively study the role of K48-polyubiquitylation.

## Data Availability

The mass spectrum raw data has been deposited to ProteomeXchange (http://www.proteomexchange.org/) with the identifiers PXD009060, PXD024048, and PXD023947 via the partner repositories iProX (https://www.iprox.org/page/HMV006.html) with identifiers IPX0001171000 and IPX0002803000, and MassIVE (https://massive.ucsd.edu/ProteoSAFe/static/massive.jsp) with identifier MSV000086795. All annotated spectra can be viewed using MS-Viewer with the search key wwpnfejbpj or via the following link: https://msviewer.ucsf.edu/prospector/cgi-bin/mssearch.cgi?report_title=MS-Viewer&search_key=wwpnfejbpj&search_name=msviewer.

## Supplemental data

This article contains supplemental data.

## Conflict of interest

The authors declare that they have no known competing financial interests or personal relationships that could have appeared to influence the work reported in this paper.
