# Supplementary material for: The Ubiquitin Interacting Motif-Like Domain of Met4 Selectively Binds K48 Polyubiquitin Chains
Source: Mol Cell Proteomics. 2021 Nov 9;21(1):100175. doi: 10.1016/j.mcpro.2021.100175 (PMC8693465; doi:10.1016/j.mcpro.2021.100175)

Supplemental Information

**Figure S1:** Nucleotide and amino acid sequences of tUBD, UIML, and UIM probes. Resides colored in green are the biotin-acceptor peptide sequence Avi tag. The 6xHis tag is colored in light blue, The SMT3 amino acid sequence is colored in orange, and the Met4 ubiquitin binding sequences are colored in magenta.

**Figure S2:** Nucleotide and amino acid sequences of UIMLx2, UIMLx3, and UIMLx4 probes. Resides colored in green are the biotin-acceptor peptide sequence Avi tag. The 6xHis tag is colored in light blue, The SMT3 amino acid sequence is colored in orange, and the Met4 ubiquitin binding sequences are colored in magenta.

**Figure S3:** In vitro characterization of UIMLx2 binding after stringent washing: (A) Clarified whole cell lysate (WCL) were bound to UIMLx2 and washed with buffer with different NaCl concentrations (150 mM, 250 mM, 500 mM, and 1000 mM) or with different (B) Urea concentrations (0 M, 0.5 M, 2 M, 4 M, and 8 M).

**Figure S4:** Sub-mitochondrial compartment localization of proteins identified as mitochondrion proteins from the DAVID analysis. MitoCarta3.0 dataset was used to identify mitochondrial localization. Left graph displays percentage localization with proteins of unknown sub-mitochondrial localization. Right graph displays only identified proteins with known sub-mitochondrial localization. (“MIM”: Mitochondrial inner membrane; “MOM”: Mitochondrial outer membrane; “IMS”: Intermembrane space)

**Figure S5:** Total ion chromatograms of the SRM analyses. The fraction 1 in figure 4 C was analyzed by selective reaction monitoring (SRM) mode to quantify different linkage types of ubiquitin chains, 2 biological replicates were performed in each SRM analysis.

**Supplemental Table 1.** Protein quantification by the LC-SRM analysis of selected peptides

**Supplemental Table 2.** Summary of all proteins identified by LC MS/MS analysis.

Figure S1


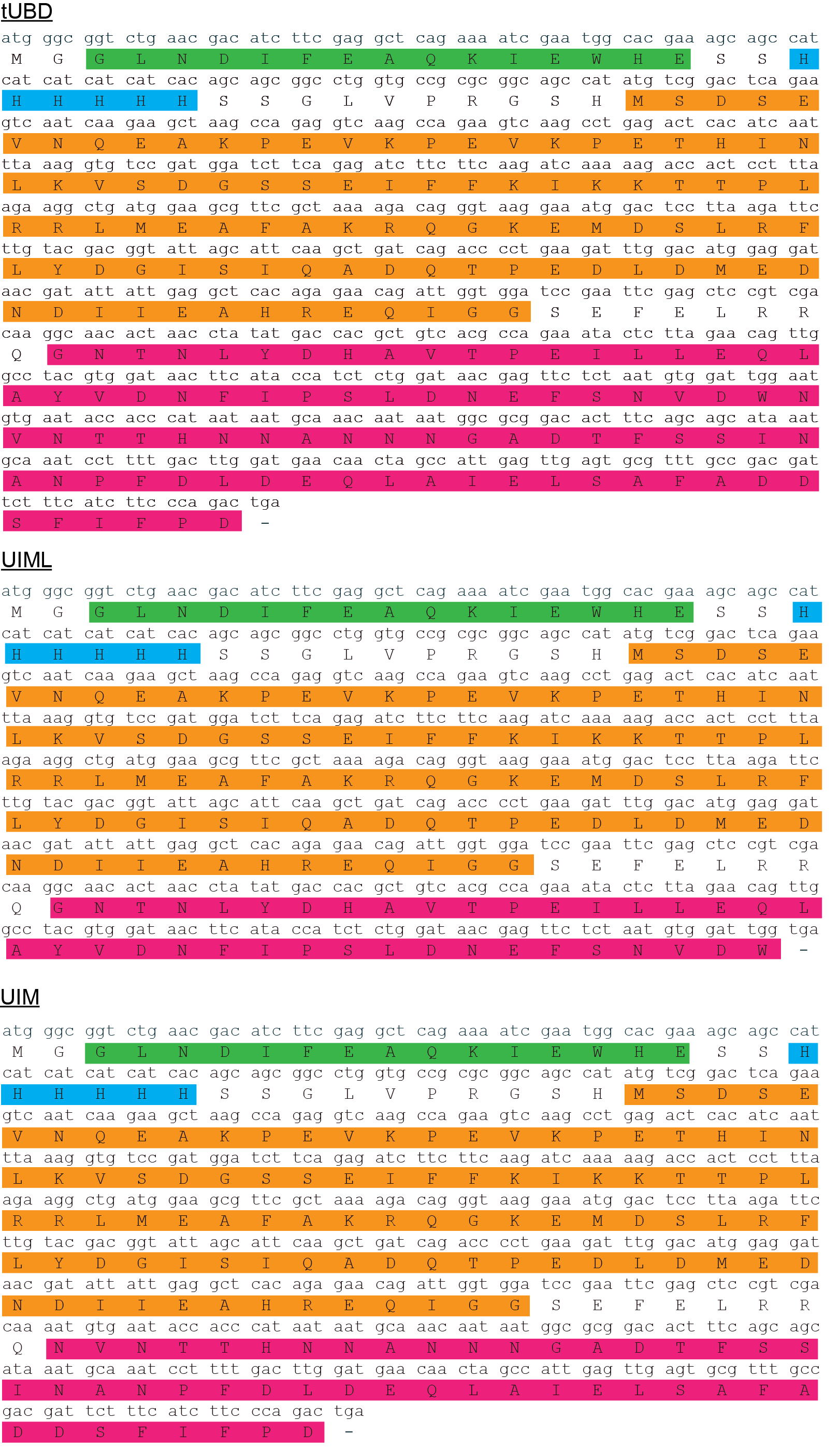


Figure S2


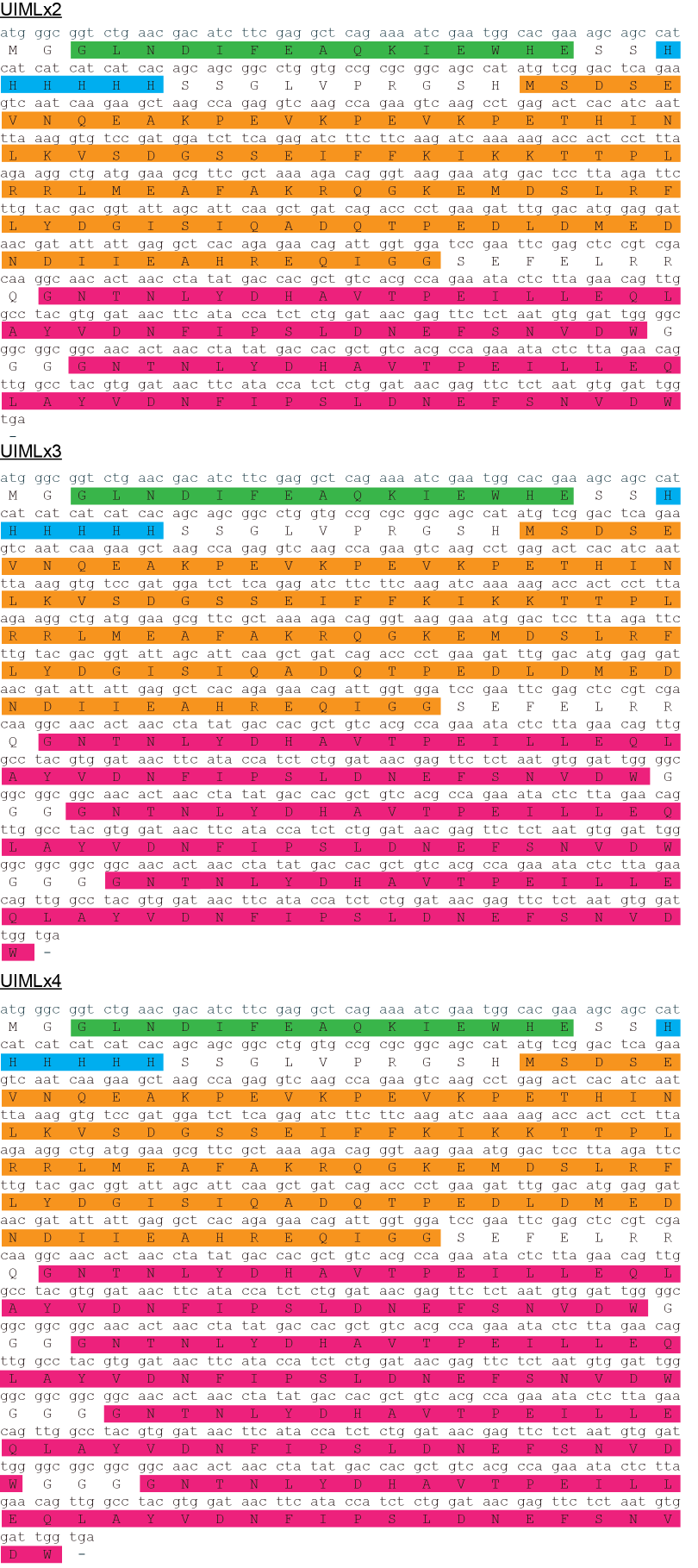


Figure S3


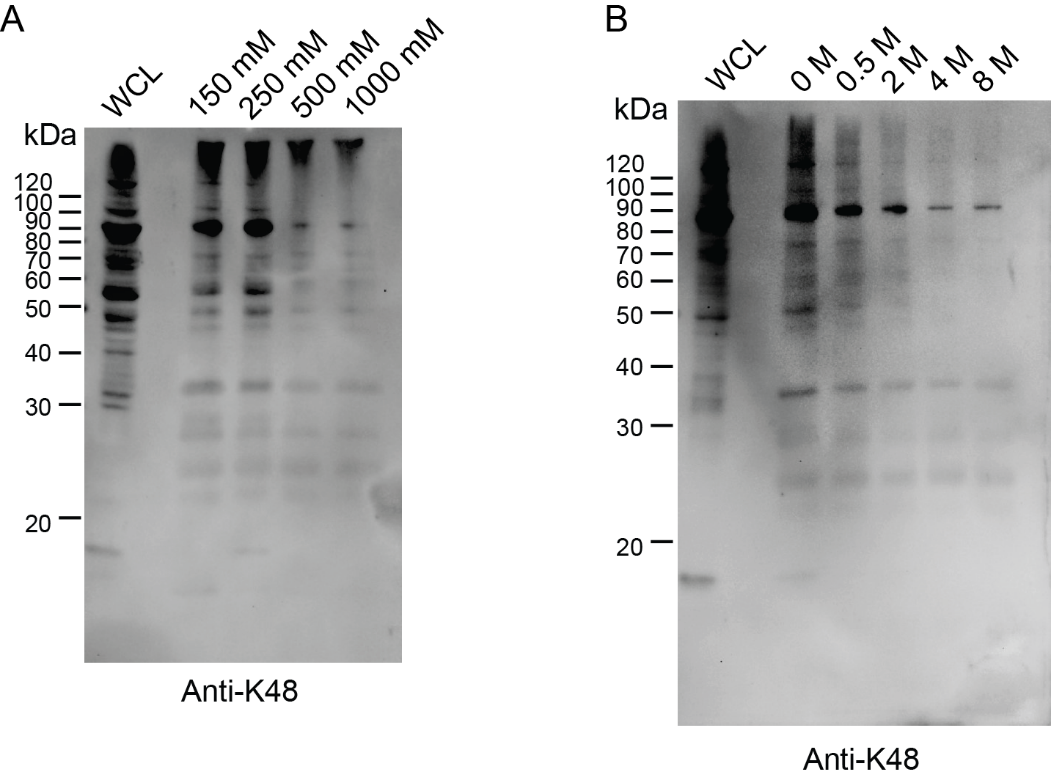


Figure S4


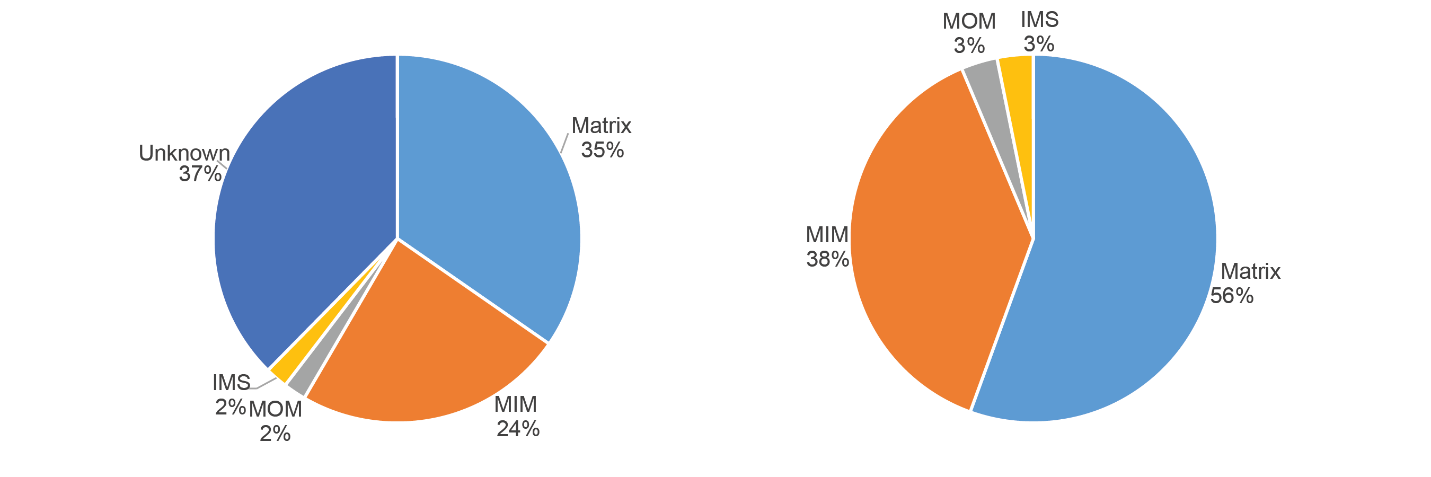


Figure S5


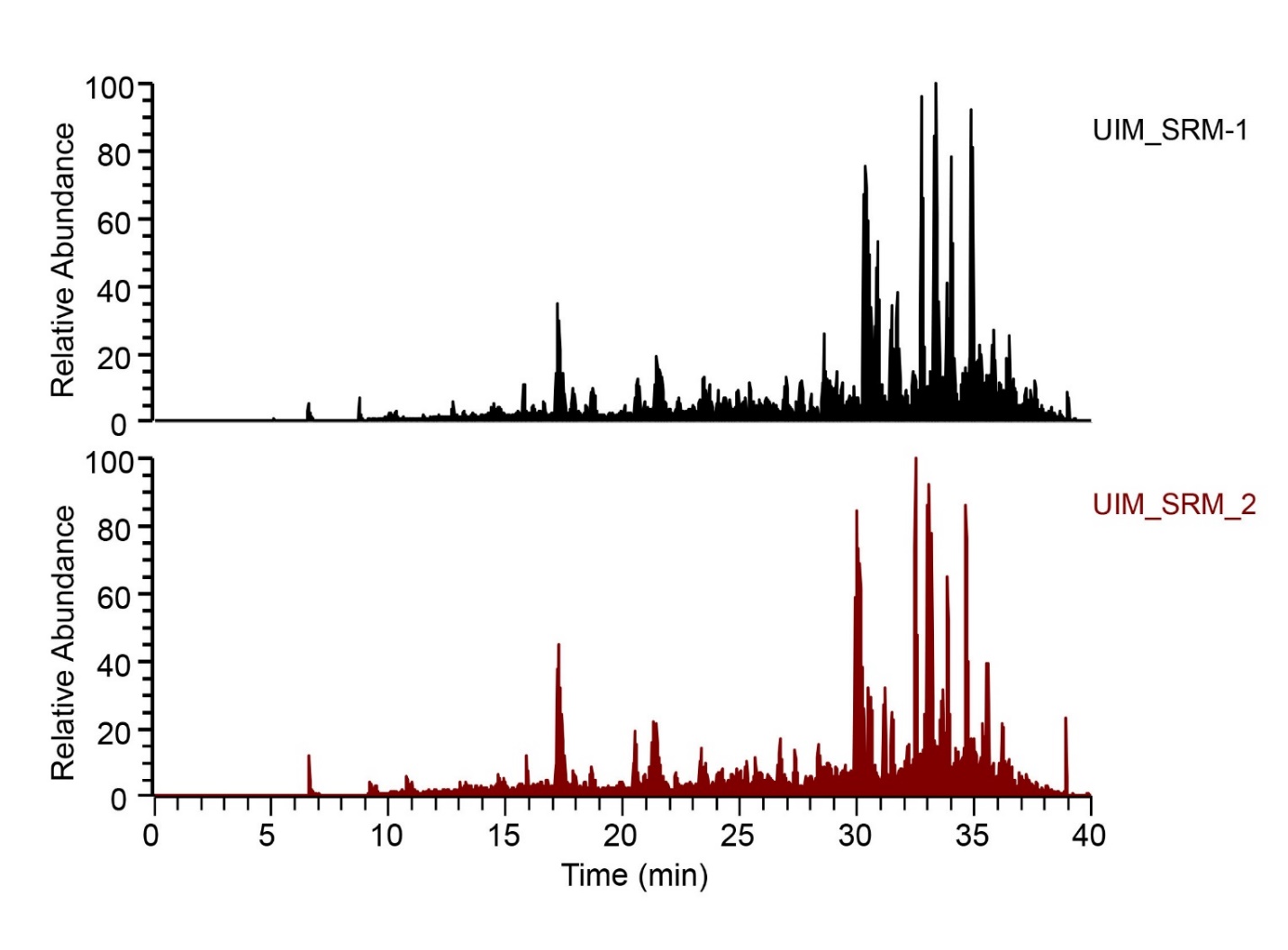

Supplement: Supplemental Figures S1–S5 [file mmc3.docx]
